# Self-reported sleep in late pregnancy in relation to birth size and fetal distress: the E Moe, Māmā prospective cohort study

**DOI:** 10.1136/bmjopen-2015-008910

**Published:** 2015-10-05

**Authors:** Laura D Howe, T Leigh Signal, Sarah-Jane Paine, Bronwyn Sweeney, Monique Priston, Diane Muller, Kathy Lee, Mark Huthwaite, Philippa Gander

**Affiliations:** 1 School of Social and Community Medicine, University of Bristol, Bristol, UK; 2 Sleep-Wake Research Centre, Massey University, Wellington, New Zealand; 3 University of California, San Francisco, California, USA; 4 University of Otago, Wellington, New Zealand

**Keywords:** SLEEP MEDICINE

## Abstract

**Objectives:**

To explore associations between features of sleep during pregnancy and adverse outcomes for the infant.

**Setting:**

E Moe, Māmā is a cohort study in Aotearoa/New Zealand that investigates self-reported sleep and maternal health in late pregnancy and the postpartum period.

**Participants:**

Women (N=633; 194 Māori) reported detailed information on their sleep duration, quality, disturbances, disorders (snoring, breathing pauses, twitching legs, restless legs) and daytime sleepiness between 35 and 37 weeks gestation.

**Outcome measures:**

Birthweight and fetal distress during labour were extracted from medical records. Associations between each sleep variable and small or large for gestational age (SGA/LGA) using customised birthweight centile or fetal distress were estimated using multinomial/logistic regression, controlling for potential confounders. Secondary analyses considered differences in associations between Māori and non-Māori women.

**Results:**

There was some indication that breathing pauses (a measure of sleep apnoea) were associated with both SGA (OR 2.8, 95% CI 0.9 to 9.0, p=0.08) and LGA (OR 2.0, 95% CI 0.7 to 5.7, p=0.20), with the association for LGA being stronger when only pregnancy-onset breathing pauses were considered (OR 3.5, 95% CI 1.3 to 9.6, p=0.01). There was also some evidence that pregnancy-onset leg twitching (OR 3.3, 95% CI 1.1 to 10.0, p=0.03) and frequent sleep disturbance due to feeling too hot or too cold (OR 1.7, 95% CI 0.9 to 3.6, p=0.13) were associated with higher risk of fetal distress. Other sleep measures, including snoring, were not associated with SGA, LGA or fetal distress. Many of the associations we observed were considerably stronger in Māori compared with non-Māori women.

**Conclusions:**

We did not find evidence of previously reported associations between snoring and SGA. Our findings tentatively suggest that self-reported breathing pauses and leg twitching in late pregnancy are associated with infant outcomes, and highlight ethnic inequalities.

**Strengths and limitations**

- This study used a broad range of measures of sleep in pregnancy, and looked at their associations with objective measures of birth weight and fetal distress, obtained from medical records.
- Importantly, we found ethnic differences in the associations, with stronger associations between sleep in pregnancy and fetal outcomes in women identifying as Maori.
- Important limitations of this study include the self-reported nature of sleep during pregnancy, and the lack of preterm births in this cohort (due to recruitment in late pregnancy).

## Background

Lower birthweight and other birth complications have considerable short-term and long-term consequences for the infant.^1–3^ While multiple risk factors for lower birthweight and birth complications have been identified,^4^ their predictive power remains modest,^5^ and there is therefore high potential benefit in identifying novel risk factors for birth complications. Despite approximately one-third of a person's life being spent asleep, the overwhelming majority of research into the potential causes of birth complications has focused on non-sleep-related factors.^6^ Sleep disturbance, changes to sleep duration and reduced sleep quality are common during pregnancy,^6–15^ and given the large body of evidence suggesting links between sleep and other health outcomes,^16–25^ investigating their role in the aetiology of birth complications is important.

There is emerging evidence that sleep changes during pregnancy may be associated with pregnancy and birth outcomes.^6^ ^26^ ^27^ Several studies have shown an association between poor sleep quality or short sleep duration during pregnancy and preterm birth,^12^ ^14^ ^28^ ^29^ with inflammatory mechanisms proposed as a potential explanation (eg, disturbed sleep leading to disruptions to normal immune processes).^13^ Some, though not all,^30^ studies have also suggested a link between sleep during pregnancy and fetal growth including identifying snoring as a risk factor for small for gestational age (SGA) infants.^29^ ^31^ The majority of the existing studies on this topic, however, have a small sample size, with only two recent studies having larger samples.^29^ ^31^ Most, though not all,^31^ studies also concentrate on preterm birth and SGA, and do not examine potential associations between sleep during pregnancy and large for gestational age (LGA) infants or other infant outcomes. Furthermore, most focus only on a small number of facets of sleep, ignoring the complex nature of sleep and the possibility for multiple dimensions to be important determinants of birth complications.

In this paper, we use data from 633 participants of a prospective cohort study of pregnant women in Aotearoa/New Zealand (NZ) to examine the associations of multiple facets of sleep in late pregnancy (sleep duration, sleep quality, sleep disturbances, sleep disorders and daytime sleepiness) with fetal distress and infant birth weight. In NZ, the Māori people are the indigenous population. Stark health inequalities exist in NZ, with Māori people suffering from worse health outcomes than non-Māori people, including higher rates of SGA infants.^32^ Therefore, as a secondary aim, we also examined whether the associations between sleep during pregnancy and infant outcomes differed between the 194 Māori participants and the 439 non-Māori participants.

## Methods

### Participants

The E Moe, Māmā: Maternal Sleep and Health in Aotearoa/NZ study (N=1186; 423 Māori and 763 non-Māori recruited October 2009–October 2011) surveyed a community sample of women during the third trimester of pregnancy (approximately 35–37 weeks gestation) who were carrying a single fetus. This sample thus excludes the majority of preterm births. Equal numbers of Māori and non-Māori were sought in recruitment.^33^

### Measurement of maternal sleep parameters

Maternal sleep in late pregnancy was assessed by self-completed questionnaire. Women were asked to report the average number of hours sleep in 24 h (including naps) during the past week, and before this pregnancy. This was categorised as short sleep (≤6 h), average sleep (>6 to <9 h) or long sleep (≥9 h). We also defined change in sleep duration from pre-pregnancy to late pregnancy, based on movement between these categories from pre-pregnancy to late pregnancy.

Pre-pregnancy and late pregnancy poor quality sleep was defined as not getting a good night's sleep on three or more nights per week, based on one of the Diagnostic and Statistical Manual of Mental Disorders, Fourth Edition (DSM-IV) criteria for insomnia. Late pregnancy sleep quality was also assessed by the General Sleep Disturbance Scale (GSDS), which was used as a total score, and separate subscale scores for sleep quality and daytime sleepiness. Each of these scores was dichotomised into problematic and non-problematic (mean item scores ≥3 or <3, respectively) sleep.^34^ ^35^

Women were asked to report the number of nights in the previous week on which a range of factors, many associated with pregnancy, had disturbed their sleep; for each factor we considered three or more disturbed nights as problematic.^36^

The Epworth Sleepiness Scale (ESS)^37^ was used to assess daytime sleepiness, with scores greater than 10 indicating excessive daytime sleepiness.

Three separate symptoms of sleep disorders were investigated: snoring, breathing pauses during sleep and leg twitching/jerking. Each symptom was considered to be present if women reported that it occurred ≥3 times per week during late pregnancy.^38^ For each of these sleep symptoms, we also defined ‘pregnancy-onset’ sleep problems as being present in women who reported the symptoms on ≥3 nights per week during late pregnancy, but on <3 nights per week pre-pregnancy. For snoring, we additionally defined ‘chronic snoring’ as snoring on ≥3 nights per week in pre-pregnancy as well as late pregnancy.

As a separate sleep disorder, restless legs was defined as a positive response to four questions: ever experiencing an urge to move legs (usually accompanied by unpleasant sensations), and whether this is worse at night, more noticeable when resting, and relieved by movement.^39^

### Measurement of infant outcomes

Infant birth weight and gender were extracted from medical records by the District Health Boards/Ministry of Health through matching on the infant's national health index number and birth date. Customised birthweight centiles were generated using the Grow software for the NZ population (using software developed by the Gestation Network, available from https://www.gestation.net/cc/about.htm. Access date 23 September 2015).^40^ This method develops birthweight centiles accounting for maternal height, weight, ethnicity, parity and the infant's sex and gestation at delivery. This method is intended to separate pathological from constitutionally small babies, and has been shown to result in stronger associations between small size at birth and perinatal mortality compared with uncustomised birthweight centiles.^41^ ^42^ We defined SGA as <10th customised centile, appropriate for gestational age (AGA) as 10–90th centile and LGA as >90th centile.

Fetal distress refers to a range of symptoms either during pregnancy or delivery that suggests the fetus may not be well. Fetal distress was defined as one or more of the following the International Classification of Diseases (ICD) 10^43^ codes being recorded in the obstetric records (ICD 10 codes shown in parentheses): maternal care for signs of hypoxia (O363), labour and delivery complicated by fetal heart rate anomaly (O680), fetal heart rate anomaly with meconium in amniotic fluid (O682), biochemical evidence of fetal stress (O683) or other evidence of fetal stress (O688).

### Measurement of potential confounders

Maternal age, parity and self-identified ethnicity were recorded in the self-completed questionnaire in late pregnancy; any woman identifying as Māori as a single response, or one of many choices of ethnicity, was classified as Māori. Everyone else was classified as non-Māori. This approach is the recommended standard for health research in NZ.^44^ Maternal smoking was categorised as ‘smokers’ (regular or occasional smokers) and ‘non-smokers’ (ex-smokers or non-smokers). Height and pre-pregnancy weight reported in the questionnaire were used to calculate pre-pregnancy body mass index (BMI; kg/m^2^). Area-level deprivation was measured using the NZ Deprivation Index 2006 (NZDep),^45^ a measure that assigns households to a 10th of deprivation based on residential address. Maternal depressive symptoms were assessed in late pregnancy using the Edinburgh Postnatal Depression Scale (EPDS).^46^ Gestational age at delivery was reported by the mothers in a questionnaire at 12 weeks postpartum.

### Statistical analysis

Analysis was restricted to participants with data on all of the measures of sleep in late pregnancy, customised birthweight centile, fetal distress, infant gender and all a priori determined potential confounders (pre-pregnancy sleep duration and number of nights good sleep, maternal age, parity, ethnicity, pre-pregnancy BMI, smoking (any vs none), NZ Deprivation Index, gestational age at delivery and maternal EPDS score).

Our primary aim was to determine the association between maternal sleep and infant outcomes in the whole population (Māori and non-Māori combined). Associations between each sleep measure and categories of customised birthweight centile were assessed using multinomial logistic regression (with AGA as the reference category) to allow for non-linear relationships between maternal sleep and infant birth weight. Associations between each sleep measure and fetal distress were assessed using logistic regression. Analyses were carried out initially with minimal adjustment, including only infant gender as a covariable in order to increase precision. Subsequent analyses included adjustment for all potential confounders, including ethnicity. Since the relationship between sleep and depression is potentially bi-directional, we conducted a sensitivity analysis removing EPDS score from the confounder-adjusted model.

Our secondary aim was to examine potential ethnic differences in the associations between maternal sleep and infant outcomes. We conducted formal tests of interaction for differences in associations between Māori and non-Māori women, and present the results of these tests and all confounder-adjusted stratified analyses in online supplementary material. As an illustration of the general pattern of ethnic differences in associations, where the point estimate suggested a strong association (OR≥2) in either ethnicity, we present ethnic stratified results in the main paper as a graph. We chose this approach rather than using a p value threshold because of low statistical power, particularly in Māori women. We used the maximum available sample size for each separate sleep measure in our ethnic stratified analyses, rather than restrict analyses to women with complete data on all sleep measures.

## Results

### Sample definition

Complete data on sleep in late pregnancy, birth weight, fetal distress and potential confounders were available for 633 women; 194 Māori women and 439 non-Māori women (46% and 57% of the original cohort). Compared with those excluded due to missing data, women included in our analysis tended to be slightly older, live in areas of lower deprivation, were less likely to identify as Māori, and more likely nulliparous (see online supplementary table S1). A detailed breakdown of the missing data for each variable is presented in online supplementary table S2. Some differences in pre-pregnancy sleep were noted, with women included in our analysis less likely to sleep for nine or more hours per night (see online supplementary table S1). Differences in sleep during late pregnancy were less evident, although there was some evidence that women included in our analysis were more likely to have poor quality sleep as measured by the GSDS and less likely to sleep for less than 6 or 9 or more hours per night compared with those excluded (see online supplementary table S3).

### Participant characteristics

Women who identified as Māori were more likely to be younger, smoke, live in more deprived areas (higher score on NZDep), have higher parity and higher EPDS depression scores. The prevalence of SGA and LGA was broadly similar in Māori and non-Māori women, but 13.9% of infants born to Māori women experienced fetal distress compared with 3.2% of infants born to non-Māori women (table 1).

**Table 1:** Participant characteristics by ethnicity

	Statistic	Mother identifies as Māori (N=194)	Mother identifies as non-Māori (N=439)	p Value*
Maternal age (years)	Mean (SD)	29.4 (6.1)	32.8 (4.8)	<0.001
NZ Deprivation Index (tenths)	Median (IQR)	7 (4 to 9)	4 (2 to 6)	<0.001
Parity	Median (IQR)	1 (0 to 2)	0 (0 to 1)	0.003
Gestational age at delivery (weeks)	Median (IQR)	40 (39 to 41)	40 (39 to 41)	0.59
Maternal BMI (kg/m^2^)	Median (IQR)	27.4 (23.0 to 30.8)	23.7 (21.5 to 26.8)	<0.001
EPDS score in late pregnancy	Median (IQR)	8 (5 to 12)	7 (4 to 11)	0.04
Maternal smoking in late pregnancy	N (%)	30 (15.5%)	12 (2.7%)	<0.001
Sleep duration before pregnancy				
Short sleep (≤6 h)	N (%)	11 (5.7%)	12 (2.7%)	
Average sleep (>6 to <9 h)		114 (58.8%)	341 (77.7%)	
Long sleep (≥9 h)		69 (35.6%)	86 (19.6%)	<0.001
3 or more poor nights’ sleep before pregnancy	N (%)	22 (11.3%)	38 (8.7%)	0.29
Birth weight	N (%)			
Small for gestational age		13 (6.7%)	43 (9.8%)	
Appropriate for gestational age		149 (76.8%)	341 (77.7%)	
Large for gestational age		32 (16.5%)	55 (12.5%)	0.22
Fetal distress	N (%)	27 (13.9%)	14 (3.2%)	<0.001

*Derived from linear regression when comparing means, non-parametric test of equality of medians, or χ^2^ tests for categorical variables.

BMI, body mass index; EPDS, Edinburgh Postnatal Depression Scale; NZ, New Zealand.

### Maternal sleep parameters

Women who identified as Māori were more likely to report a short (≤6 h) or long (≥9 h) pre-pregnancy sleep duration compared with non-Māori women (table 1). Ethnic differences in late pregnancy sleep were also evident, including a higher prevalence in Māori women of daytime sleepiness; sleep disturbance due to back, neck or joint pain, feeling too hot or cold, not being able to get comfortable, or not being able to sleep; long sleep duration; and leg twitching (table 2).

**Table 2:** Sleep in late pregnancy by ethnicity

	Mother identifies as Māori (N=194)	Mother identifies as non-Māori (N=439)	p Value for comparison
Poor sleep quality			
3 or more nights poor sleep	163 (84.0%)	364 (82.9%)	0.73
GSDS total	167 (86.1%)	380 (86.6%)	0.87
GSDS quality subscale	165 (85.1%)	366 (83.4%)	0.60
GSDS daytime sleepiness subscale	112 (57.7%)	202 (46.0%)	0.01
Sleep disturbances on three or more nights			
Going to the bathroom	177 (91.2%)	396 (90.2%)	0.68
Pain in back/neck/joints	147 (75.8%)	269 (61.3%)	<0.001
Dreams	83 (42.8%)	139 (31.7%)	0.01
Nightmares	21 (10.8%)	29 (6.6%)	0.07
Heartburn	72 (37.1%)	162 (36.9%)	0.77
Nasal congestion	50 (25.8%)	118 (26.9%)	0.77
Leg cramps	62 (32.0%)	108 (24.6%)	0.05
Contractions	31 (16.0%)	42 (9.6%)	0.02
Feeling too hot or too cold	112 (57.7%)	204 (46.5%)	0.01
Thinking or worrying about things	95 (49.0%)	191 (43.5%)	0.20
Baby moving about	127 (65.5%)	241 (54.9%)	0.01
Other children	60 (30.9%)	101 (23.0%)	0.04
Just cannot get comfortable	149 (76.8%)	271 (61.7%)	<0.001
Just cannot get to sleep	103 (53.1%)	166 (37.8%)	<0.001
Disturbed by partner	50 (25.8%)	98 (22.3%)	0.34
Sleep duration			
Short sleep (≤6 h)	57 (29.4%)	118 (26.9%)	
Average sleep (>6 to <9 h)	90 (46.4%)	247 (56.3%)	
Long sleep (≥9 h)	47 (24.2%)	74 (16.9%)	0.04
Change in sleep duration between pre-pregnancy and late pregnancy*			
Increased	28 (14.4%)	62 (14.1%)	
No change	83 (42.8%)	210 (47.8%)	
Decreased	83 (42.8%)	167 (38.0%)	0.47
Sleep disorders			
Snoring	47 (24.2%)	104 (23.7%)	0.88
Chronic snoring	21 (10.8%)	29 (6.6%)	0.07
Pregnancy-onset snoring	22 (11.3%)	82 (18.7%)	0.02
Breathing pauses	11 (5.7%)	13 (3.0%)	0.10
Pregnancy-onset breathing pauses	8 (4.1%)	12 (2.7%)	0.36
Leg twitching	36 (18.6%)	32 (7.3%)	<0.001
Pregnancy-onset leg twitching	9 (4.6%)	26 (5.9%)	0.52
Restless legs	28 (14.4%)	81 (18.5%)	0.22
Excessive daytime sleepiness (ESS≥10)	60 (30.9%)	96 (21.9%)	0.02

Values are number (percent); p values are obtained from χ^2^ tests.

*Defined as movement between the defined categories of short sleep (≤6 h), average sleep (>6 and <9 h) and long sleep (≥9 h).

ESS, Epworth Sleepiness Scale; GSDS, General Sleep Disturbance Scale.

Poor sleep quality was common in all women in late pregnancy, with 84% of Māori and 83% of non-Māori women reporting three or more nights of poor quality sleep in late pregnancy (table 2) compared with 11.3% and 8.7% retrospective pre-pregnancy reports (table 1). All results were similar with and without adjustment for EPDS score, so only results with this variable included in the set of confounders are presented.

### Maternal sleep in relation to SGA infants

In analysis of Māori and non-Māori women combined, there was no strong evidence that poor sleep quality or other sleep parameters were associated with SGA (see online supplementary table S4 for minimally adjusted results and table 3 for confounder-adjusted results). Long sleep duration (≥9 h) was weakly associated with SGA (OR 1.6 after adjustment for confounders, with 95% CI 0.8 to 3.2, p=0.19). Among reasons for sleep disturbance, only nasal congestion showed a suggestion of association with SGA (OR 1.8 with 95% CI 1.0 to 3.2, p=0.06). Snoring, pregnancy-onset snoring, chronic snoring, leg twitching, pregnancy-onset leg twitching and restless legs showed no association with SGA. There was a weak association between breathing pauses and SGA (OR 2.8 with 95% CI 0.9 to 9.0, p=0.08), but this association was not observed for pregnancy-onset breathing pauses.

**Table 3:** Confounder-adjusted associations between sleep in late pregnancy and customised infant birthweight centile (N=633)

Measure of sleep	Birth size using customised birthweight centiles for the NZ population		
	Small for gestational age (<10th centile)	Appropriate for gestational age	Large for gestational age (>90th centile)
	N=56	N=490	N=87
Poor sleep quality			
3 or more nights poor sleep	1.0 (0.4 to 2.0) p=0.89	1 (ref)	0.9 (0.5 to 1.6) p=0.61
GSDS total	1.1 (0.5 to 2.7) p=0.77	1 (ref)	1.0 (0.5 to 2.0) p=0.99
GSDS quality subscale	1.0 (0.4 to 2.1) p=0.90	1 (ref)	0.7 (0.4 to 1.3) p=0.23
GSDS daytime sleepiness subscale	1.3 (0.7 to 2.3) p=0.45	1 (ref)	1.2 (0.7 to 1.9) p=0.52
Sleep disturbances on three or more nights per week			
Going to the bathroom	1.1 (0.4 to 3.0) p=0.82	1 (ref)	1.5 (0.6 to 3.6) p=0.39
Pain in back/neck/joints	1.1 (0.6 to 2.0) p=0.81	1 (ref)	1.0 (0.6 to 1.6) p=0.87
Dreams	0.8 (0.4 to 1.5) p=0.46	1 (ref)	1.5 (0.9 to 2.4) p=0.10
Nightmares	0.7 (0.2 to 2.4) p=0.57	1 (ref)	0.5 (0.2 to 1.5) p=0.21
Heartburn	0.6 (0.3 to 1.1) p=0.09	1 (ref)	0.8 (0.5 to 1.3) p=0.29
Nasal congestion	1.8 (1.0 to 3.2) p=0.06	1 (ref)	1.0 (0.6 to 1.7) p=0.95
Leg cramps	0.6 (0.3 to 1.2) p=0.14	1 (ref)	1.2 (0.7 to 2.0) p=0.55
Contractions	1.0 (0.4 to 2.6) p=0.96	1 (ref)	1.3 (0.6 to 2.6) p=0.48
Feeling too hot or too cold	0.9 (0.5 to 1.6) p=0.81	1 (ref)	1.2 (0.8 to 2.0) p=0.40
Thinking or worrying about things	1.1 (0.6 to 1.9) p=0.85	1 (ref)	1.6 (1.0 to 2.6) p=0.08
Baby moving about	1.3 (0.7 to 2.4) p=0.34	1 (ref)	1.3 (0.8 to 2.1) p=0.29
Other children	1.1 (0.5 to 2.3) p=0.76	1 (ref)	1.1 (0.6 to 2.1) p=0.79
Just cannot get comfortable	1.2 (0.7 to 2.3) p=0.48	1 (ref)	1.2 (0.7 to 2.0) p=0.53
Just cannot get to sleep	0.7 (0.4 to 1.3) p=0.27	1 (ref)	1.4 (0.9 to 2.2) p=0.19
Disturbed by partner	0.2 (0.1 to 0.7) p=0.01	1 (ref)	1.4 (0.8 to 2.3) p=0.26
Sleep duration			
Short sleep (≤6 h)	0.9 (0.4 to 1.8) p=0.72	1 (ref)	1.1 (0.6 to 1.9) p=0.76
Average sleep (>6 to <9 h)	1 (ref)	1 (ref)	1 (ref)
Long sleep (≥9 h)	1.6 (0.8 to 3.2) p=0.19	1 (ref)	1.1 (0.6 to 2.0) p=0.83
Change in sleep duration between pre-pregnancy and late pregnancy*			
Increased	1.4 (0.6 to 3.2) p=0.47	1 (ref)	1.4 (0.7 to 2.9) p=0.29
No change	1 (ref)	1 (ref)	1 (ref)
Decreased	0.8 (0.4 to 1.5) p=0.50	1 (ref)	1.3 (0.7 to 2.2) p=0.37
Sleep disorders			
Snoring	1.2 (0.6 to 2.4) p=0.52	1 (ref)	1.2 (0.7 to 2.0) p=0.58
Chronic snoring	1.6 (0.6 to 4.0) p=0.32	1 (ref)	1.7 (0.8 to 3.7) p=0.14
Pregnancy-onset snoring	0.8 (0.4 to 1.8) p=0.58	1 (ref)	1.1 (0.6 to 2.1) p=0.75
Breathing pauses	2.8 (0.9 to 9.0) p=0.08	1 (ref)	2.0 (0.7 to 5.7) p=0.21
Pregnancy-onset breathing pauses	1.7 (0.4 to 7.9) p=0.52	1 (ref)	3.5 (1.3 to 9.6) p=0.01
Les twitching	1.4 (0.6 to 3.5) p=0.45	1 (ref)	1.0 (0.5 to 2.2) p=0.99
Pregnancy-onset leg twitching	0.2 (0.0 to 1.9) p=0.18	1 (ref)	0.6 (0.2 to 2.1) p=0.42
Restless legs	0.9 (0.4 to 2.0) p=0.86	1 (ref)	1.5 (0.8 to 2.6) p=0.19
Excessive daytime sleepiness (ESS≥10)	0.8 (0.4 to 1.6) p=0.52	1 (ref)	1.4 (0.8 to 2.4) p=0.19

Coefficients are ORs with 95% CIs from logistic regressions, adjusted for infant gender, ethnicity (Māori or non-Māori), NZ Deprivation Index, maternal age, parity, BMI, EPDS score, smoking (any vs none), pre-pregnancy sleep duration and number of poor nights’ sleep.

*Defined as movement between categories of short sleep (≤6 h), average sleep (>6 and <9 h) and long sleep (≥9 h).

BMI, body mass index; EPDS, Edinburgh Postnatal Depression Scale; ESS, Epworth Sleepiness Scale; GSDS, General Sleep Disturbance Scale; NZ, New Zealand.

### Maternal sleep in relation to LGA infants

There was a strong association between pregnancy-onset breathing pauses and LGA (OR 3.5 with 95% CI 1.3 to 9.6, p=0.01). Other maternal sleep measures were either not associated with LGA or only weakly associated (see online supplementary table S4 for minimally adjusted results and table 3 for confounder-adjusted results).

### Maternal sleep in relation to fetal distress

There was no evidence of associations between sleep quality, daytime sleepiness, sleep duration or changes in sleep duration and fetal distress (table 4) for both the pre-pregnancy and late pregnancy reports. Sleep disturbance due to feeling too hot or too cold had a higher odds of fetal distress (OR 1.7 with 95% CI 0.9 to 3.6, p=0.13) but did not reach statistical significance. Pregnancy-onset leg twitching was associated with a threefold higher odds of fetal distress (OR 3.3 with 95% CI 1.1 to 10.0, p=0.03).

**Table 4:** Associations between sleep in late pregnancy and fetal distress (N=633)

Measure of sleep	OR and 95% CI for fetal distress	
	Minimally adjusted association*	Adjusted association†
Poor sleep quality		
3 or more nights poor sleep	0.8 (0.4 to 1.9) p=0.65	0.8 (0.3 to 1.9) p=0.61
GSDS total	0.7 (0.3 to 1.7) p=0.48	0.7 (0.3 to 1.8) p=0.45
GSDS quality subscale	1.4 (0.5 to 3.7) p=0.50	1.4 (0.5 to 3.9) p=0.52
GSDS daytime sleepiness subscale	1.3 (0.7 to 2.5) p=0.38	1.0 (0.5 to 2.2) p=0.92
Sleep disturbances on three or more nights per week		
Going to the bathroom	0.6 (0.2 to 1.4) p=0.22	0.6 (0.2 to 1.5) p=0.25
Pain in back/neck/joints	1.4 (0.7 to 2.9) p=0.31	1.0 (0.5 to 2.2) p=0.93
Dreams	1.5 (0.8 to 2.8) p=0.24	1.2 (0.6 to 2.4) p=0.62
Nightmares	1.3 (0.4 to 3.8) p=0.64	0.9 (0.3 to 2.9) p=0.87
Heartburn	1.0 (0.5 to 1.9) p=0.98	1.1 (0.5 to 2.1) p=0.88
Nasal congestion	1.0 (0.5 to 2.1) p=0.98	0.9 (0.4 to 2.0) p=0.86
Leg cramps	0.7 (0.3 to 1.6) p=0.42	0.6 (0.2 to 1.3) p=0.17
Contractions	0.4 (0.1 to 1.6) p=0.19	0.3 (0.1 to 1.4) p=0.14
Feeling too hot or too cold	2.0 (1.0 to 3.9) p=0.04	1.7 (0.9 to 3.6) p=0.13
Thinking or worrying about things	1.2 (0.6 to 2.2) p=0.66	1.0 (0.5 to 2.0) p=0.90
Baby moving about	1.1 (0.6 to 2.1) p=0.76	1.0 (0.5 to 2.1) p=0.96
Other children	0.7 (0.3 to 1.5) p=0.37	1.4 (0.5 to 3.7) p=0.56
Just cannot get comfortable	0.7 (0.4 to 1.3) p=0.29	0.5 (0.2 to 1.0) p=0.04
Just cannot get to sleep	1.2 (0.6 to 2.2) p=0.62	0.9 (0.4 to 1.8) p=0.68
Disturbed by partner	0.9 (0.4 to 2.0) p=0.85	0.8 (0.4 to 1.8) p=0.63
Sleep duration	1 (ref)	1 (ref)
Short sleep (≤6 h)	1.4 (0.7 to 2.8) p=0.42	1.6 (0.7 to 3.5) p=0.26
Average sleep (>6 to <9 h)	1 (ref)	1 (ref)
Long sleep (≥9 h)	1.4 (0.6 to 3.1) p=0.47	1.2 (0.5 to 3.0) p=0.66
Change in sleep duration between pre-pregnancy and late pregnancy‡		
Increased	1.4 (0.5 to 3.9) p=0.47	1.4 (0.5 to 4.1) p=0.55
No change	1 (ref)	1 (ref)
Decreased	1.8 (0.9 to 3.7) p=0.09	1.5 (0.7 to 3.4) p=0.28
Sleep disorders	1 (ref)	1 (ref)
Snoring	1.2 (0.6 to 2.4) p=0.65	1.1 (0.5 to 2.4) p=0.79
Chronic snoring	1.7 (0.6 to 4.5) p=0.30	1.5 (0.5 to 4.2) p=0.48
Pregnancy-onset snoring	0.2 (0.1 to 1.0) p=0.06	0.3 (0.1 to 1.2) p=0.09
Breathing pauses	0.6 (0.1 to 4.8) p=0.65	0.5 (0.1 to 4.0) p=0.50
Pregnancy-onset breathing pauses	0.8 (0.1 to 6.0) p=0.81	0.8 (0.1 to 6.7) p=0.86
Leg twitching	1.1 (0.4 to 3.0) p=0.79	0.5 (0.2 to 1.6) p=0.28
Pregnancy-onset leg twitching	2.6 (0.9 to 7.0) p=0.07	3.3 (1.1 to 10.0) p=0.03
Restless legs	1.2 (0.5 to 2.6) p=0.71	1.1 (0.5 to 2.5) p=0.89
Excessive daytime sleepiness (ESS≥10)	1.1 (0.5 to 2.3) p=0.75	1.0 (0.5 to 2.2) p=0.93

Coefficients are ORs and 95% CIs for fetal distress (n=41) compared with no fetal distress.

*Adjusted for infant gender.

†Adjusted for infant gender, ethnicity (Māori or non-Māori), NZ Deprivation Index, maternal age, parity, BMI, EPDS score, gestational age at delivery, smoking (any vs none), pre-pregnancy sleep duration and number of poor nights’ sleep.

‡Defined as movement between the defined categories of short sleep (≤6 h), average sleep (>6 and <9 h) and long sleep (≥9 h).

BMI, body mass index; EPDS, Edinburgh Postnatal Depression Scale; ESS, Epworth Sleepiness Scale; GSDS, General Sleep Disturbance Scale; NZ, New Zealand.

### Ethnic differences in the associations between maternal sleep and infant outcomes

**Figure 1:**
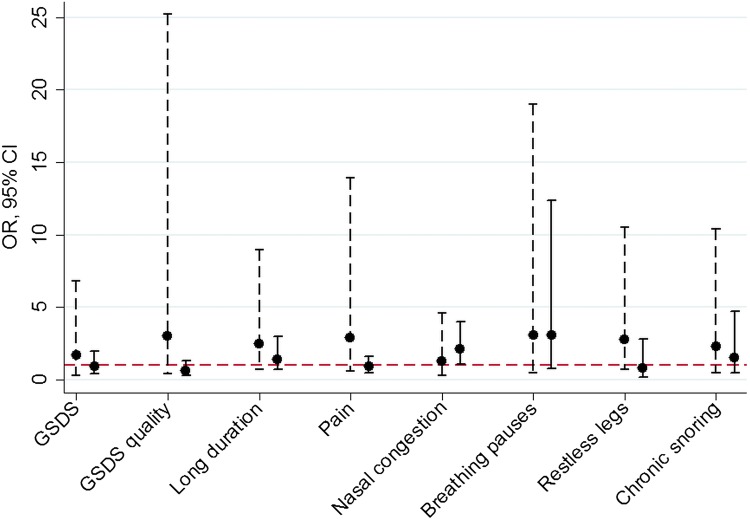
Associations between sleep in late pregnancy and small for gestational age stratified by mother's Māori versus non-Māori ethnicity. Legend: ORs and 95% CIs are shown for the association between measures of sleep in late pregnancy and small for gestational age (<10th customised birthweight centile) for Māori (dashed lines) and non-Māori (solid lines) women. The red dashed line indicates the null value (OR=1). Associations are shown for sleep measures where the OR≥2 in either ethnicity. GSDS is the General Sleep Disturbance Scale; GSDS quality is the sleep quality subscale of the GSDS; long duration is sleep duration ≥9 h; pain refers to disturbed sleep on three or more nights per week due to back, neck or shoulder pain; nasal congestion refers to disturbed sleep on three or more nights per week due to nasal congestion; breathing pauses refers to breathing pauses on three or more nights per week; restless legs is defined as having an urge to move legs (usually accompanied by unpleasant sensations) that is worse at night, more noticeable when resting and relieved by movement; chronic snoring is snoring on three or more nights a week both pre-pregnancy and in late pregnancy.

**Figure 2:**
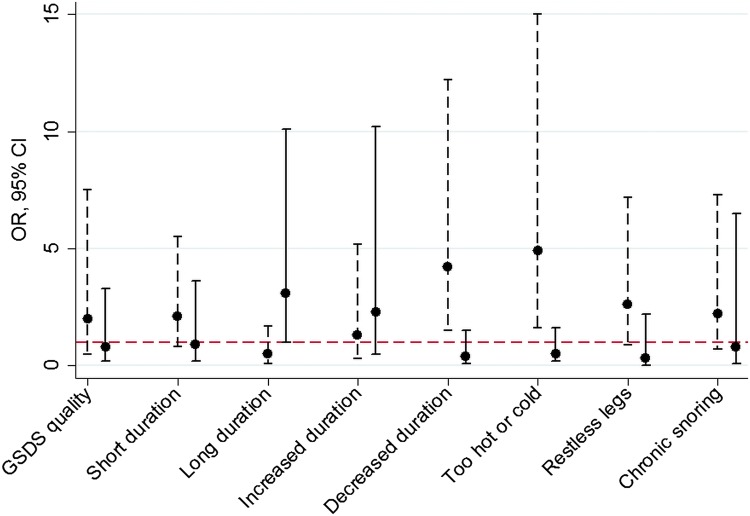
Associations between sleep in late pregnancy and fetal distress stratified by mother's Māori versus non-Māori ethnicity. Legend: ORs and 95% CIs are shown for the association between measures of sleep in late pregnancy and fetal distress for Māori (dashed lines) and non-Māori (solid lines) women. Associations are shown for sleep measures where the OR≥2 in either ethnicity. GSDS quality is the quality subscale of the General Sleep Disturbance Scale; short duration is sleep duration ≤6 h; long duration is sleep duration ≥9 h; increased duration means moving from short (≤6 h) to average (>6 to <9 h) or average to long (9≥ h) duration of sleep from pre-pregnancy to late pregnancy; decreased duration is the reverse of this; too hot or cold is sleep disturbance on three or more nights per week due to feeling too hot or too cold; restless legs is defined as having an urge to move legs (usually accompanied by unpleasant sensations) that is worse at night, more noticeable when resting and relieved by movement; chronic snoring is snoring on three or more nights a week both pre-pregnancy and in late pregnancy.

There was statistical evidence of Māori/non-Māori differences in associations between sleep in late pregnancy and birth outcomes for a small number of sleep measures (see online supplementary tables S5–7). Considering all sleep measures, regardless of whether the interaction test provided statistical evidence for ethnic differences, the general pattern was for associations to be stronger in Māori women (figures 1 and 2). For example, the OR for fetal distress due to feeling too hot or too cold was 4.9 in Māori women (95% CI 1.6 to 15.0, p<0.001) compared with 0.5 in non-Māori women (95% CI 0.2 to 1.6) and the OR for fetal distress due to restless legs was 2.6 in Māori women (95% CI 0.9 to 7.2, p=0.07) compared with 0.3 in non-Māori women (95% CI 0.0 to 2.2, p=0.22).

## Discussion

In this cohort of women in the last trimester of pregnancy, we observed that self-reported poor sleep quality and sleep disturbances were common, with 84% of Māori women and 83% of non-Māori women reporting three or more nights of poor quality sleep per week. Many women experienced reductions in sleep duration and quality from pre-pregnancy to 35–37 weeks gestation, and problematic sleep tended to be more common in women identifying as Māori.

### Maternal sleep in relation to infant birthweight and fetal distress

This study is the first to report associations between a large range of maternal sleep measures and infant outcomes. We found little evidence of associations between most of the sleep measures we investigated and the risk of a baby being born SLA or LGA. There was weak evidence that experiencing breathing pauses on three or more nights per week in late pregnancy (a measure of sleep apnoea) was associated with higher risk of both SGA and LGA, which was robust to adjustment for self-reported maternal BMI. For SGA, this association was not present for pregnancy-onset breathing pauses (ie, women who experience this symptom in late pregnancy but did not experience it pre-pregnancy). For LGA, the association was observed for both current breathing pauses and pregnancy-onset breathing pauses, but was strongest for pregnancy-onset. Our results provide some evidence that pregnancy-onset leg twitching and frequent sleep disturbance due to feeling too hot or too cold are associated with higher risk of fetal distress. The association between breathing pauses and birth size, particularly LGA, could to some degree reflect gestational diabetes due to known associations between obstructive sleep apnoea (OSA) and glucose intolerance and between gestational diabetes and macrosomia. In this study, 5% (N=5) of mothers with LGA infants reported having diabetes, compared with 2% (N=15) of mothers with AGA infants. Owing to the small numbers, it was not possible to further evaluate the role of diabetes in the associations we observe.

Several other studies have examined sleep in pregnancy in relation to infant birth weight. Snoring has been postulated as a determinant of fetal growth and hence infant birth weight. A prospective cohort study of 1673 women in the USA found that chronic snoring (snoring both before and during pregnancy) was associated with a higher risk of the infant being SGA (OR 1.65, 95% CI 1.02 to 2.66), but there was no association between snoring and LGA.^31^ Among 1091 women in a prospective cohort in Crete, Greece, severe snoring (women who reported snoring frequently or always) was associated with higher risk of low birth weight (<2.5 kg) and with customised birth weight below the 10th centile; relative risks after adjusting for various confounders (including maternal BMI) were 2.6 (95% CI 1.2 to 5.4) and 2.0 (1.0 to 3.8).^29^ We did not replicate these findings in our analyses; we found no evidence of an association between snoring (current snoring on three or more nights per week, pregnancy-onset snoring on three or more nights per week, or chronic snoring defined as snoring on three or more nights per week both pre-pregnancy and in late pregnancy) and either SGA or LGA. The reasons for the difference between our results and those of previous studies are unclear. It is possible that our sample size was insufficient to detect the association, or that the lack of association in our cohort is due to population differences. Recruitment into the E Moe, Māmā cohort took place in late pregnancy (most women were between 35 and 37 weeks gestation at completion of the initial questionnaire); thus the number of preterm births in our sample is extremely small. Other studies included a higher percentage of preterm infants, and this may explain some of the differences in results. Definitions of snoring also differ slightly between studies.

In addition to the studies on snoring and birth weight, a small study involving 14 pregnant women with OSA and 27 controls found that OSA was associated with a customised birth weight less than the 10th centile or a reduction in customised centile of more than 33% between the 32-week ultrasound and delivery.^47^ Our analysis, which included a much larger number of women but from a population-based sample, rather than clinical cases, found weak evidence of association between frequent (3 or more nights per week) breathing pauses during sleep and neither SGA nor LGA. Given the wide CIs in our analysis, this finding requires replication in other large population-based studies, and examination in studies with objective sleep measures, which may shed light on the mechanisms.

We found no evidence that sleep duration was associated with SGA or LGA. A previous study of 200 women found that sleep duration was only weakly related to birth weight, with mean birth weight 3516.9 g in women who slept more than 8 h in the third trimester, 3502.2 g in those who slept 6–8 h and 3442.4 g in those who slept less than 6 h.^48^ This study, however, considered birth weight as a continuum, and so is not directly comparable to our analysis of SGA and LGA.

Fetal distress has, to our knowledge, not been considered as a potential consequence of poor sleep in pregnancy. It is possible that our observed associations with leg twitching and sleep disturbance due to feeling too hot or too cold may be due to underlying medical conditions, although we could find no clear evidence of this using the available (self-reported, free-text) medical data. Since this is the first study to examine the associations between sleep in pregnancy and fetal distress, the observed associations with leg twitching and sleep disturbance due to feeling too hot or cold require confirmation in other large studies.

### Ethnic differences in the associations between maternal sleep and infant outcomes

Stark inequities in health exist between the Māori and non-Māori populations of NZ, for both sleep^49^ ^50^ and infant^32^ outcomes, and it is thus imperative to examine ethnic differences in health research. The women identifying as Māori in this study reported worse sleep during pregnancy than the non-Māori women, for example, having a higher prevalence of short sleep duration and many sleep disturbances. The reasons for this difference are unclear and require further exploration, for example, in qualitative studies. For many of the associations we examined, the associations of sleep during pregnancy with birthweight or fetal distress were either stronger in women identifying as Māori, or present only in Māori women. For example, global GSDS scores; the GSDS quality subscale; long sleep duration; sleep disturbance due to back, neck or joint pain; and restless legs demonstrated stronger associations with SGA in Māori women, and the GSDS quality subscale, short sleep duration, decreased sleep duration from pre-pregnancy to late pregnancy, sleep disturbance due to feeling too hot or too cold, and restless legs demonstrated stronger associations with fetal distress in Māori women. It therefore appears that for many of the associations we see in the combined population, these are being driven by the Māori women.

The reasons underlying the stronger associations between maternal sleep and infant outcomes in Māori women are uncertain, but are likely to be complex and multifactorial. One possibility is that the self-reported sleep measures may capture different underlying processes for the Māori and non-Māori women (ie, women in these groups may interpret the questions slightly differently, or place different emphases on sleep as an aspect of health and well-being). The questionnaires used in this study have not been validated in a Māori population. A similar consideration is that some of the confounding variables we adjusted for may act differently in Māori and non-Māori women. A third potential explanation is that we have created binary variables indicating problematic/non-problematic sleep for each sleep measure, and it is possible that for each sleep measure, Māori women have on average worse levels than non-Māori women. Further research, including qualitative studies to explore potential differences in the interpretation of the sleep questionnaire and studies with objective measures of sleep, is required to disentangle these potential explanations. It is noteworthy that intervention studies designed to help with maternal sleep have shown greater improvement among more disadvantaged women.^35^

The finding of stronger associations between maternal sleep and infant outcomes emphasises an important need to focus on the sleep and other health concerns of Māori pregnant women in order to reduce inequalities in health for both the mother and her infant.^44^ ^49^ A multilevel approach to improve sleep may help all women, and address ethnic inequalities. This may involve better/more education for lead maternity carers and other health providers about the importance of sleep, the existence of ethnic inequalities, and how these might impact on mother and infant well-being; screening and identification of sleep problems in pregnancy; appropriate interventions/strategies for reducing and/or managing symptoms and monitoring the mother and infant; and policy-level interventions around funding and planning will be critical. Issues of ethnic inequalities in access to antenatal care are also likely to be important.

### Strengths and limitations

In this analysis, we have utilised data from a large prospective cohort study with data on many potential confounding factors. A key strength of our approach is the availability of a broad array of measurements of sleep in late pregnancy, allowing us to test whether multiple facets of sleep have different consequences for birth outcomes. A consequence of this, however, is that we have conducted multiple statistical tests and there is a possibility that the associations we observe are false positives. Replication in other large cohort studies is essential to clarify the role of sleep parameters on birth outcomes, and where associations are replicated and confirmed, the underlying biological mechanisms need exploration in studies with objective sleep measures. The availability of data on pre-pregnancy and pregnancy snoring, leg twitching and breathing pauses enabled us to study differential consequences of pregnancy-onset sleep disorders, as emphasised by previous studies.^31^ Using self-reported maternal BMI means that there is a possibility of residual confounding due to measurement error in this variable. We were able to adjust for pre-pregnancy sleep duration and sleep quality in order to reduce confounding of our associations of interest, and to examine whether change in sleep duration from pre-pregnancy to late pregnancy was an important determinant of birth outcomes. However, it would also be interesting in future studies to examine the dynamic nature of sleep across the different stages of pregnancy. While our data on sleep in late pregnancy were self-reported and assessed at a single time point during late pregnancy, this is the case in most other studies on this topic.^12^ ^31^ Future studies using objective measures of sleep such as actigraphy, particularly with repeated measures across different stages of pregnancy, will make a valuable contribution to this literature. We observed important differences in associations between Māori and non-Māori women. The non-Māori group represents a mixture of ethnicities, including other groups that suffer a health disadvantage in NZ, such as Pacific Island people.^51^ ^52^ Thus, we may have underestimated the magnitude of Māori and non-Māori differences in the associations of interest, and we were unable to examine other ethnic differences due to small numbers in each group.

## Conclusion

This analysis provides a novel angle to the emerging literature on the topic of sleep in pregnancy and birth complications, considering a broader range of sleep measures than previous studies, and evaluating ethnic inequalities in the associations between maternal sleep and infant outcomes. We did not replicate previous findings that snoring is associated with SGA, but our results tentatively suggest that breathing pauses (a measure of sleep apnoea) may be associated with both SGA and LGA, and that pregnancy-onset leg twitching and frequent sleep disturbance due to feeling too hot or too cold are associated with higher risk of fetal distress, particularly for women identifying as Māori. Initiatives to improve the sleep of pregnant women, and reduce ethnic inequalities, may improve infant outcomes.
